# Supplementary material for: Electron Tomography as a Tool to Study SARS-CoV-2 Morphology
Source: Int J Mol Sci. 2024 Nov 1;25(21):11762. doi: 10.3390/ijms252111762 (PMC11547116; doi:10.3390/ijms252111762)
Supplement: Supplementary file 1 [file ijms-25-11762-s001.zip › Legends of Supplementary Materials.pdf]

Supplementary Materials

Electron Tomography as a Tool to Study SARS-CoV-2 Morphology

Wu et al.

Supplementary Video S1. Representative 3D ET images of HIV-1 in 293T cells reconstituted from the epoxy resin-embedded samples. The left and right panels are Y rotation and Z slice position images of the HIV-1 virion, respectively.

Supplementary Video S2. 3D ET images of TFV 17D particles found in the intracellular vacuole of infected Vero cells.
